# Population-based validation of a German version of the Brief Resilience Scale

**DOI:** 10.1371/journal.pone.0192761

**Published:** 2018-02-13

**Authors:** Andrea Chmitorz, Mario Wenzel, Rolf-Dieter Stieglitz, Angela Kunzler, Christiana Bagusat, Isabella Helmreich, Anna Gerlicher, Miriam Kampa, Thomas Kubiak, Raffael Kalisch, Klaus Lieb, Oliver Tüscher

**Affiliations:** 1 Deutsches Resilienz Zentrum (DRZ), University Medical Center Mainz, Mainz, Germany; 2 Department of Psychiatry and Psychotherapy, University Medical Center Mainz, Mainz, Germany; 3 Health Psychology, Institute for Psychology, Johannes Gutenberg University Mainz, Mainz, Germany; 4 Department of Psychology, University of Basel, Basel, Switzerland; 5 Neuroimaging Center (NIC) Mainz, University Medical Center Mainz, Mainz, Germany; Universitat Wien, AUSTRIA

## Abstract

Smith and colleagues developed the Brief Resilience Scale (BRS) to assess the individual ability to recover from stress despite significant adversity. This study aimed to validate the German version of the BRS. We used data from a population-based (sample 1: n = 1.481) and a representative (sample 2: n = 1.128) sample of participants from the German general population (age ≥ 18) to assess reliability and validity. Confirmatory factor analyses (CFA) were conducted to compare one- and two-factorial models from previous studies with a method-factor model which especially accounts for the wording of the items. Reliability was analyzed. Convergent validity was measured by correlating BRS scores with mental health measures, coping, social support, and optimism. Reliability was good (α = .85, ω = .85 for both samples). The method-factor model showed excellent model fit (sample 1: χ2/df = 7.544; RMSEA = .07; CFI = .99; SRMR = .02; sample 2: χ2/df = 1.166; RMSEA = .01; CFI = 1.00; SRMR = .01) which was significantly better than the one-factor model (Δχ^2^(4) = 172.71, p < .001) or the two-factor model (Δχ^2^(3) = 31.16, p < .001). The BRS was positively correlated with well-being, social support, optimism, and the coping strategies active coping, positive reframing, acceptance, and humor. It was negatively correlated with somatic symptoms, anxiety and insomnia, social dysfunction, depression, and the coping strategies religion, denial, venting, substance use, and self-blame. To conclude, our results provide evidence for the reliability and validity of the German adaptation of the BRS as well as the unidimensional structure of the scale once method effects are accounted for.

## Introduction

Over the past decades, the concept of psychological resilience has stimulated a plethora of research in different fields including the psychological, medical and neurobiological sciences [1–6]. The term resilience refers to the phenomenon that many people maintain mental health or only temporally become mentally ill despite significant adversity [2,7–10]. Following that definition, resilience is viewed as an outcome and not as static personality trait [4].

With regard to the assessment of resilience, several measures have been developed to assess putative resilience factors, that is, the majority of those instruments are based either on a trait-oriented approach (e.g., Dispositional Resilience Scale [DRS]; [11]) or focus on measuring the availability of resources and protective factors to maintain or regain mental health despite significant adversities (e.g., Connor-Davidson Resilience Scale [CD-RISC]; [12]). In order to assess resilience itself, that is, the individual ability to recover from stress despite significant adversity (e.g., chronic stressors or adverse life events) more closely, Smith and colleagues [13] developed the Brief Resilience Scale (BRS).

The BRS [13] is a short six-item measure that assesses the ability to “bounce back” from stress on a five-point Likert scale. It showed good psychometric properties with high internal consistency and retest-reliability. The unidimensional scale has been shown to be related to other resilience measures (e.g. CD-RISC; [12], personal characteristics (e.g., optimism assessed by the Life Orientation Test-Revisited [LOT-R]; [14]), health outcomes (e.g., depression assessed by the Hospital anxiety and depression scale [HADS]; [15]), coping styles (e.g., active coping assessed by the Brief COPE; [16]) and social relationships (e.g., social support assessed by the Interpersonal Support Evaluation List [ISEL]; [17]) and to distinguish between healthy and clinical samples (fibromyalgia patients, patients in cardiac rehabilitation) [13]. With regard to health outcomes, the predictive value of the BRS exceeded other resilience scales that are based on a trait definition of resilience (e. g., Ego Resiliency Scale; [18]) or primarily assess protective factors (e.g., CD-RISC; [12]). Compared to other resilience scales, the psychometric properties of the BRS were rated high in terms of internal consistency (i.e., Cronbach’s α ranging from .80–90) and convergent (i.e., correlation of BRS score ≥ .30 with conceptually similar measure) as well as discriminant predictive validity (i.e., correlation of BRS score ≥ .30 with theoretically distinct measure) [19,20].

Up to now, the BRS has been translated into Dutch, Malaysian, Portuguese, and Spanish. The validation studies show adequate psychometric qualities. The Dutch version (BRSnl) was validated on a sample of rehabilitation unit residents (n = 40) [21]. Here resilience was conceptualized as absence of depression or anxiety, measured by the Hospital Anxiety Depression Scale (HADS) [15]. They used Receiver Operating Curve (ROC) analyses to assess accuracy of the six items and the total score in detecting the condition with a score of seven or less on the HADS [15]. The area under curve (AUC) for the BRSnl was .84 [95% CI: .73 - .92]. Internal consistency was assessed by unstandardized Cronbach’s α. For test-retest reliability, BRSnl mean scores at baseline and BRSnl mean scores at four-week follow-up were compared. They found a Cronbach’s α of .83 and an Intraclass Correlation Coefficient (ICC) of .94 [22]. Congruent validity was assessed by correlating the BRSnl and the Dutch version of the Resilience Scale [23]. Moderate correlations were reported (at baseline: r = .35; follow-up: r = .50). To evaluate construct validity, they used the Positive and Negative Affect Scale (PANAS) [24], the Life Orientation Test (LOR-R) [14], the visual analog scale (VAS) for pain [25] and the HADS. High scores on the BRSnl were related to higher positive affect, lower HADS scores and lower negative affect in the linear mixed models. It was positively correlated with optimism (r = .51) and negatively correlated with pessimism (r = -.13).

The Malaysian version was validated on n = 120 international students [26]. The authors assessed the internal consistency using Cronbach’s α and the factor structure of the scale by principal component analysis (PCA). They reported a Cronbach’s α of .93. In the PCA they found a single factor (eigenvalue = 4.41) accounting for 73.54% of the total variance. The respective factor loadings ranged from .82 to .91.

The Portuguese version was validated in two adult samples (sample 1: n = 171; sample 2: n = 232) [27]. In sample 1, the authors conducted a PCA and analyzed internal consistency (Cronbach’s α). Moreover, they calculated correlations of the BRS scores and hypothetically related constructs using the Positivity Scale [28] and the Flourishing scale [29]. After excluding one item (item 5 “I usually come through difficult times with little trouble”), the results of the PCA provided evidence for a one-factor solution (eigenvalue = 2.58) which accounted for 43% of the total variance. The authors did not discuss the conceptual basis for item exclusion. The reliability of the scale was adequate (Cronbach’s α = .76). They found weak correlations between BRS scores and positivity or flourishing, respectively. In sample 2, they conducted a CFA to confirm the one-factor structure. The results of the CFA also supported a one-factor solution and yielded acceptable fit indices for a five-item version (χ2/df = 9.553; CFI = .98; TLI = .96; RMSEA = .06 [90% CI = 0 - .112]). They also calculated correlations with personality traits using a shortened version of the Big Five Inventory (BFI) [30] to further assess the validity of the BRS. To provide evidence on the convergent validity of the scale, they computed the average variance extracted (AVE). The AVE (.47) was below the recommended threshold of .50. They observed weak positive correlations between BRS and extraversion (r = .19), openness (r = 0.17) and agreeableness (r = .15) and a negative correlation with neuroticism (r = -.45). In addition, they calculated the composite reliability (CR) and obtained an adequate value (CR = .81).

So far, the largest validation study was conducted for the Spanish version of the BRS [31]. Here, a total of n = 620 adults was examined by combining several heterogeneous samples (e.g., parents of oncology outpatient children, oncology patients or the general population). The authors calculated the internal consistency and retest-reliability of the scale and conducted a CFA. In contrast to De Holanda Coelho and colleagues [27], the authors included two first-order factors (one for the positively and one for the negatively worded items) in the model to account for a potential effect of positively and negatively worded items [32–34]. The CFA provided evidence for a one-factor solution with adequate fit indices for the six-item version (χ2/df = 2.36; SRMR = .036; GFI = .980; CFI = .984; IFI = .984; RMSEA = .067). The Spanish version also showed adequate reliability (Cronbach’s α = .83, test-retest ICC = .69). In addition, to analyze convergent and concurrent validity, correlations between BRS scores and the Connor-Davison Resilience Scale (CD-RISC) [12], the Perceived Stress Scale (PSS) [35], the Modified Differential Emotions Scale (mDES) [36], the Situational Coping Scale for Adults (SCSA) [37] and the Personality Factors for Resilience (PFR) were calculated. The mDES, the SCSA and the PFR was developed by the authors based on existing scales. They found positive and statistical significant correlations between the BRS and the CD-RISC, positive emotions (mDES), problem centered coping (SCSA), sense of mastery, sense of relatedness and emotional reactivity from the PFR scale; they reported negative correlations with stress (PSS), negative emotions (mDES) and emotion centered coping (SCSA), presuming adequate convergent and concurrent validity of the BRS. They also assessed predictive validity by calculating the correlations between BRS and HADS [15], the Davidson Trauma Scale (DTS) [38] and the Posttraumatic Growth Inventory (PTGI) [39]. Negative and statistical significant correlations between the BRS and HADS as well as DTS were reported.

To validate the German version of the BRS, we used two large population-based adult samples (sample 1: n = 1.481; sample 2: n = 1.128) to assess the reliability of the scale and its factor structure. Based on the findings of previous studies [26,27,31], we tested one- and two-factorial models and also a method factor model to account for potential wording effects in order to identify the model with the best model fit. To examine convergent and discriminant validity, we analyzed the latent correlations among BRS and measures of mental health, well-being, optimism, and social support as well as correlations with coping styles. In line with Smith and colleagues [13], we expect negative correlations between the BRS and somatic symptoms, anxiety/insomnia, social dysfunction, severe depression and dysfunctional coping styles (e.g., behavioral disengagement, denial, self-blame or substance use) and positive correlations between BRS and well-being, social support, optimism and functional coping styles (e.g., active coping, planning or positive reframing). Discriminant validity was established by comparing the latent correlations with average variance extracted (AVE) of the BRS. We expect that the BRS explains more than half of the variance.

There is evidence on potential sociodemographic differences in BRS scores. With regard to gender and age differences, Smith and colleagues [13] reported higher BRS scores in male cardiac patients than female cardiac patients. No difference was found in the samples of undergraduate students [13]. Rodruiguez-Rey and colleagues [31] found higher BRS scores for men. With regard to age, Smith and colleagues [40] reported a positive correlation between age and BRS scores. In the original paper [13], the assessment of age-related differences was not reported. Rodriguez-Rey and colleagues [31] also found lower levels of BRS scores for participants between the age of 20 and 30 years compared to 31 years and older.

The aim of this study was to assess the reliability and validity of the German translation of the BRS and to investigate potential differences related to gender or age.

## Materials and methods

### Participants

#### Sample 1

We analyzed data from the Gutenberg Brain Study (GBS). The GBS is a population-based sample of healthy adults aged 18 to 75 years, living in a city located in the southwest of Germany. The data collection started in February 2014 and is still ongoing. In order to assess the reliability and validity of the BRS, we administered the scale to all GBS participants who were recruited between May 2015 and December 2016 (n = 1.481).

To recruit the GBS sample, potential participants were randomly selected via official local residents’ registers. Based on that information, potential participants were contacted by letter and invited to take part in the study. For contact initiation, we sent out an initial invitation followed by a second invitation after fourteen days for the non-responders. Those who agreed to take part in the study were screened for study eligibility by trained staff using a structured telephone interview. The inclusion and exclusion criteria were assessed by standardized questions during the telephone interview. The inclusion criteria were: age 18 to 75 years, normal or corrected vision, and sufficient knowledge of the German language. The exclusion criteria were: lifetime diagnosis of schizophrenia or bipolar disorder, organic mental disorders, or substance dependence syndromes. We further excluded participants with known learning disabilities, serious neurological disorders (e. g., tumors in the central nervous system), or regular use of prescribed psychoactive medications in the past six months. Following verbal consent, potential participants who met the study criteria were provided with study materials, including a Patient Information Leaflet, a consent form to sign and return to obtain written consent, a questionnaire booklet. Those participants who reported exclusion criteria in the questionnaire but not in the screening interview were also excluded. The GBS study protocol was approved by the ethics committee at the Rhineland-Palatinate state chamber of physicians (No 837.085.13, 8770-F).

#### Sample 2

This sample includes participants of a representative survey of the German population. The data was collected by The Institut für Demoskopie Allensbach (IfD Allensbach) between August and September 2016. IfD Allensbach conducted face-to-face interviews with n = 1.128 people with the minimum age of 18 years. The individuals were selected as they met criteria of the quota sample based on the German official statistics. Thus the data can be generalized to the German population with normal three percent of statistical uncertainty within representative surveys. In order to ensure informed consent, participants were informed about the objectives of the study, procedures of data storage and protection and their right to withdraw from the study at any point in time. They were informed that their participation is voluntary. Verbal consent was obtained to ensure anonymity.

The interviewers had a standardized questionnaire and could answer further questions for example if there were uncertainties. The study was approved by the ethics committee at the Rhineland-Palatinate state chamber of physicians (No 837.209.14, 9448F) and the participation in the survey was voluntarily.

### Study design

We used a cross-sectional, observational study design by administering the German translation of the BRS (sample 1 and 2) together with other questionnaires assessing theoretically related outcomes and constructs (sample 1 only).

### Materials

#### Brief Resilience Scale

The items of the BRS were independently translated from English into German by three German native speakers with extensive English language proficiency. In accordance with the original version, the German translation of the BRS consists of six items (original English version: item 1 “I tend to bounce back quickly after hard times”, item 2 “I have a hard time making it through stressful events”, item 3 “It does not take me long to recover from a stressful event”, item 4 “It is hard for me to snap back when something bad happens”, item 5 “I usually come through difficult times with little trouble”, item 6 “I tend to take a long time to get over set-backs in my life”; German translation: item 1”Ich neige dazu mich nach schwierigen Zeiten schnell zu erholen’, item 2”Es fällt mir schwer, stressige Situationen durchzustehen”, item 3”Ich brauche nicht viel Zeit, um mich von einem stressigen Ereignis zu erholen”, item 4”Es fällt mir schwer zur Normalität zurückzukehren, wenn etwas Schlimmes passiert ist”, item 5”Normalerweise überstehe ich schwierige Zeiten ohne größere Probleme”, item 6”Ich brauche tendenziell lange, um über Rückschläge in meinem Leben hinwegzukommen”). The items are rated on a five-point Likert scale (1 = strongly disagree, 2 = disagree, 3 = neutral, 4 = agree, 5 = strongly agree or 1 = stimme überhaupt nicht zu, 2 = stimme eher nicht zu, 3 = neutral, 4 = stimme eher zu, 5 = stimme vollkommen zu, respectively). Item 1, 3, and 5 are positively phrased; items 2, 4, and 6 are negatively phrased. To evaluate the questionnaire, the coding of the negatively phrased items is reversed in order to calculate the mean of the six items [13]. To examine whether the validity of the German translation was comparable to the results reported in the original version [13], we also assessed health-related outcomes, coping, social relationships, and other personal characteristics using the following established and validated questionnaires in sample 1.

#### Health-related outcomes

General Health Questionnaire (GHQ-28) [41]. The GHQ-28 is a self-report screening questionnaire measuring the intensity of symptoms encountered in mental disorders, mainly depression and anxiety disorders as well as insomnia. It comprises 28 items that are scored on a four-point Likert scale (0 = not at all; 3 = much more than usual) and comprises four subscales representing four dimensions of mental health symptoms: somatic symptoms, anxiety and insomnia, social dysfunction, and severe depression. A high total score in the GHQ-28 questionnaire indicates a high number of symptoms. In the present study, we administered the previously validated German version of the GHQ-28 [42].

WHO-Five Well-Being Index (WHO-5) [43]. The WHO-5 is a five-item self-administered questionnaire measuring the well-being on a six-point Likert scale (0 = at no time; 5 = all of the time). Higher scores on the unidimensional scale indicate better well-being. The previously validated German version [44] was used in this study.

#### Coping styles

Brief COPE [16]. The Brief COPE was included to assess coping styles of the participants. The self-report inventory consists of 28 items measuring 14 different coping strategies (active coping, planning, positive reframing, acceptance, humor, religion, using emotional support, using instrumental support, self-distraction, denial, venting, substance use, behavioural disengagement, and self-blame) on a four-point Likert scale (1 = not at all; 4 = very much). Higher scores indicate higher levels of the respective coping strategies. In the current study, the German version of the instrument [45] was administered.

#### Social relationships

Oslo Social Support Scale (OSS-3)[46]. The self-rating questionnaire consists of three items measuring general social support by asking the number of close friends, involvement and interest from other people, and getting practical support from neighbors. The measure uses one four-point (1 = none; 4 = six or more) and two different five-point scales (1 = no concern and interest; 5 = a lot of concern and interest). A higher degree of social support is indicated by higher scores in the OSS-3. We used the German version [47] of the instrument.

#### Other personal characteristics

Optimism/Pessimism Scale (SOP2) [48]. The questionnaire consists of two items assessing self-rated optimism and pessimism. The questionnaire uses a seven-point Likert scale (1 = not at all optimistic; 7 = very optimistic). To calculate the mean, the reverse scoring of the item pessimism is used.

### Data analysis

Primary analyses in the study comprised the analysis of the factor structure, reliability, and validity of the BRS. To validate the factor structure of the BRS, we used confirmatory factor analyses with maximum likelihood, covariance matrices, and the Satorra-Bentler method of estimation to account for potential non-normality in the distribution of the data. Given the factor structure in prior research [26,27,31], we fitted three models: (1) a one-factor model of general resilience, (2) a two-factor model with one factor for positively worded items (item 1, item 3, item 5) and one factor for negatively worded items (item 2, item 4, item 6), and (3) a two-factor model with general resilience (item 1, item 2, item 3, item 4, item 5, item 6) and a method factor (item 2, item 4, item 6) reflecting the positive and negative wording of the items. In line with Hu and Bentler [49], we assessed and compared the fit of these models using the chi-square-test, the Root Mean Square Error of Approximation (RMSEA), the Comparative Fit Index (CFI), and the Standardized Root Mean Squared Residual (SRMR). Although a general consensus on acceptable levels of fit indices is still missing [50] a value of .08 or less for RMSEA, .95 or more for CFI, and .06 or less for the SRMR is considered as an acceptable fit [49]. We used the likelihood ratio (LR) test to analyze the fit of the three models. A statistical significant result means that the fit of the alternative model is better than of the prior (null) model.

For the reliability, we did not only calculate Cronbach’s α, but also estimated the reliability using the composite reliability (McDonald’s omega ω) as suggested previously [51]. Convergent validity between the BRS and health-related measures, coping styles, social relationships, and optimism was determined by analyzing the standardized covariance between the BRS and relevant constructs, i.e., mental health (GHQ-28), well-being (WHO-5), social support (OSS-3), optimism (SOP2) and coping styles (brief COPE), whereas discriminant validity was established by comparing these latent correlations with AVE of the BRS [52]. An AVE greater than .50 is considered acceptable in that it indicates that more than 50% of the variance in a measure is due to the hypothesized construct [53].

In a last step, we analyzed the association between BRS scores and sociodemographic variables using latent means analysis. Here, we analyzed latent mean differences for the selected model for gender or age, respectively. Age was transformed to a categorical variable (group 1: < 40 years, group 2: 40–59, group 3: ≥ 60 years). Index categories were ‘men’ (for gender) and ‘age group < 40 years’ (for age). We also tested the measurement invariance of the model between groups (men vs. women or age group 1, 2 or 3, respectively).

Statistical significance of effects was determined by p values of p < .05 or by 95% confidence intervals (CI). All analyses were conducted in Stata Version 1 or the software package R (R 3.2.3), respectively.

## Results

### Sample characteristics

Table 1 provides an overview of the sample characteristics. In sample 1, around two third of the participants were women (63%), almost one third was younger than 30 years (31%) and almost three quarters had a higher educational background (72%). In sample 2, around half of the participants were women (52%), the age distribution was balanced between ages 18 to 79 (between 13 and 19% per group), only a few participants were older than 80 (3%). Less than half of the participants had a higher educational background (42%). The mean BRS score in samples 1 (M = 3.58) was higher than in sample 2 (M = 3.37).

**Table 1:** Descriptive statistics of sample 1 and sample 2.

	Sample 1 (n = 1,481)		Sample 2 (n = 1,128)	
Variable	n	Percentage	n	Percentage
**Gender**				
Female (%)	929	62.09	596	52.48
**Age (M / SD)**	1473	42.56 (16.52)	1118	51.05 (17.90)
18–29 years (%)	458	31.09	178	15.92
30–39 years (%)	244	16.56	141	12.61
40–49 years (%)	241	16.36	202	18.07
50–59 years (%)	234	15.89	214	19.14
60–69 years (%)	195	13.24	166	14.85
70–79 years (%)	101	6.86	182	16.28
80 + years (%)	-	-	35	3.13
Not reported	8	-	-	-
**Formal education**				
Ongoing (%)	20	1.35	-	-
No (%)	7	0.47	14	1.26
Up to 9 years (%)	156	10.53	239	21.47
Up to 10 years (%)	205	13.84	393	35.31
Up to 12 years (%)	1060	71.57	467	41.96
Others (%)	27	1.82	-	-
Not reported	6	0.42	-	-
**BRS score** (M/SD)	1481	3.58 (.76)	1128	3.37 (.95)

Note: M = mean; SD = standard deviation (listed in parentheses); BRS = Brief Resilience Scale.

### Factor structure

As indicated in Table 2, the method-factor model (model 3) fit the data significantly better than the one-factor model (model 1) (Δχ^2^(4) = 172.71, p < .001) or the two-factor model (model 3) (Δχ^2^(3) = 31.16, p < .001) in both samples. The overall model fit of the method-factor model (model 3) was excellent as indicated by the CFI and SRMR and good as indicated by the RMSEA (sample 1: χ2/df = 7.544; RMSEA = .07; CFI = .99; SRMR = .02; sample 2: χ2/df = 1.166; RMSEA = .01; CFI = 1.00; SRMR = .01). Moreover, factor loadings were satisfactory in both samples (Table 3), with factors consistently exceeding a magnitude of .50. Both samples showed similar factor loadings and item 2 (“Es fällt mir schwer, stressige Situationen durchzustehen / I have a hard time making it through stressful events”) demonstrated the lowest factor loading of the BRS. Given the excellent fit of the method-factor model (model 3), we opted to analyze the reliability, validity and group differences in the following for this model only.

**Table 2:** Results from CFAs and model comparisons.

Sample	Model	n	Chi^2	df	p	RMSEA	CFI	SRMR	AIC	LR test
1	(1) One Factor	1481	160.87	9	< .001	.11	.94	.05	22360.35	
	(2) Two Factors (positively or negativelyworded items)	1481	68.62	8	< .001	.07	.98	.03	22220.80	141.55***
	(3) Two Factors (method factor)	1481	37.72	5	< .001	.07	.99	.02	22195.64	31.16***
2	(1) One Factor	1128	81.50	9	< .001	.09	.97	.04	19022.95	
	(2) Two Factors (positively or negativelyworded items)	1128	15.58	8	.046	.03	1.00	.02	18932.18	92.77***
	(3) Two Factors (method factor)	1128	5.83	5	.323	.01	1.00	.01	18926.11	12.07**

Notes. Chi^2^ = Chi squared; df = degrees of freedom; RMSEA = Root Mean Square Error of Approximation; CFI = Comparative Fit Index; SRMR = Standardized Root Mean Squared Residual; AIC = Akaike Information criterion; LR = Likelihood Ratio; RMSEA und Chi^2 indicate Satorra-Bentler-scaled values., LR test = Likelihood-ratio test compared to the prior model; Model 1 = one-factor model of general resilience (items 1–6); Model 2 = two-factor model with one factor for positively worded items (item 1, item 3, item 5) and one factor for negatively worded items (item 2, item 4, item 6); Model 3 = two-factor model of general resilience (items 1–6) and a method factor reflecting the positively and negatively worded items

* = p < .05.

** < .01.

*** < .001.

**Table 3:** BRS item content, descriptive statistics, factor loadings, and inter-item correlations.

BRS item	Item (German / English)	M	SD	Factor Loading	Item 2	Item 3	Item 4	Item 5	Item 6
1	Ich neige dazu, mich nach schwierigen Zeiten schnell zu erholen / I tend to bounce back quickly after hard times	3.74 / 3.65	0.96 / 1.22	.78 / .76	.39 / .45	.62 / .59	.44 / .44	.57 / .59	.59 / .55
2	Es fällt mir schwer, stressige Situationen durchzustehen / I have a hard time making it through stressful events (R)	2.45 / 2.67	1.04 / 1.25	.58 / .63		.36 / .44	.46 / .44	.44 / .43	.47 / .50
3	Ich brauche nicht viel Zeit, um mich von einem stressigen Ereignis zu erholen / It does not take me long to recover from a stressful event	3.54 / 3.35	1.03 / 1.29	.67 / .71			.34 / .40	.50 / .54	.47 / .50
4	Es fällt mir schwer zur Normalität zurückzukehren, wenn etwas Schlimmes passiert ist / It is hard for me to snap back when something bad happens (R)	2.61 / 2.85	1.05 / 1.29	.63 / .65				.41 / .45	.59 / .59
5	Normalerweise überstehe ich schwierige Zeiten ohne größere Probleme / I usually come through difficult times with little trouble	3.61 / 3.46	0.98 / 1.21	.72 / .72					.55 / .50
6	Ich brauche tendenziell lange, um über Rückschläge in meinem Leben hinwegzukommen / I tend to take a long time to get over set-backs in my life (R)	2.37 / 2.73	1.04 / 1.27	.78 / .75					

Notes. M = mean; SD = standard deviation; R = reverse-coded; Data for Sample 1 and Sample 2 are separated by a forward slash. Factor loadings are standardized.

### Reliability

Reliability using Cronbach’s α showed good reliability, with α = .85 in both samples. The composite reliability was ω = .85 in both samples. This can be interpreted as the proportion of a scale’s variance due to a unidimensional factor [54].

### Convergent and discriminant validity

To examine convergent and discriminant validity, we analyzed the latent correlations among BRS and measures of health-related measures, social support, and optimism (Table 4) in sample 1. The BRS was negatively correlated with all four GHQ-28 subscales (somatic symptoms, anxiety/insomnia, social dysfunction, and severe depression) and positively correlated with well-being, social support, and optimism, indicating sufficient convergent validity. To assess discriminant validity, we calculated the AVE for BRS and compared it with the squared latent correlations between the constructs. Discriminant validity is established for any of the pairs if AVE is larger than the squared correlation of another construct. The AVE for BRS was acceptable (.55) and none of the squared latent correlations, ranging from .08 (social dysfunction) to .29 (somatic symptoms, well-being), exceeded the AVE.

**Table 4:** Correlations between the BRS and other measures in the German version of the BRS (sample 1) compared to original findings by Smith and colleagues [13].

	BRS (Sample 1, n = 1,481)		BRS (Smith et al., 2008, Sample 1–4)
Measure	Coef.	[95% CI]	Coef.
**Health-related outcomes**			
Somatic symptoms	-.47***	[-.53, -.42]	-.28* to -.50**
Anxiety/insomnia	-.45***	[-.50, -.40]	-.46** to -.60**
Social dysfunction	-.27***	[-.32, -.21]	n. a.
Severe depression	-.41***	[-.46, -.36]	-.41** to -.66**
Well-being	.54***	[.49, .59]	n. a.
**Social support**	.37***	[.31, .44]	.27* to .40**
**Optimism**	.49***	[.44, .54]	.45** to .69***
**Coping styles**			
Active coping	.17***	[.10., .24]	.31* to .41**
Planning	-.05	[-.03, -.12]	.27**, .42**
Positive reframing	.30***	[23., .36]	.31* to .41**
Acceptance	.19***	[.12, .26]	.22 to .43**
Humor	.21***	[.14, .28]	.08 to .32**
Religion	-.13**	[-.20, -.06]	.08, .16
Using emotional support	.04	[-.03, .11]	.10 to .16
Using instrumental support	-.05	[-.12, .03]	-.12 to .33*
Self-distraction	-.06	[-.13, .01]	-.26, .07
Denial	-.24***	[-.31, -.18]	-.32* to -.53**
Venting	-.14***	[-.21, -.07]	-.14 to .16
Substance use	-.13***	[-.20, -.06]	-.45** to -.06
Behavioral disengagement	-.02	[-.10, -.05]	-.52**, -.39**
Self-blame	-.31***	[-.38, -.25]	-.47** to -.27**

Notes.

* = p < .05.

** < .01.

*** < .001.

Due to the large number of coping styles with only two indicators, the full model with the fourteen coping styles subscales did not converge. Thus, we present the zero-order correlations between resilience and the coping styles in Table 4. The BRS was positively correlated with active coping, positive reframing, acceptance, and humor and negatively correlated with religion, denial, venting, substance use, and self-blame.

### Association between sociodemographic variables and BRS scores

To assess gender- and age-related differences in BRS scores, we first tested for measurement invariance of the method-factor model for gender and age, respectively. The results showed that strong measurement invariance could only be assessed for both gender and age in sample 2 (χ^2^(5) = 3.94, p = .558 and χ^2^(11) = 16.52, p = .123, respectively) but not in sample 1 (χ^2^(5) = 18.48, p = .002 and χ^2^(11) = 21.05, p = .033, respectively). Thus, we only only compared the BRS means between the gender and age groups in sample 2. For gender, we found that women reported lower BRS scores than men (M_Diff_ = -.47, SE_M_ = 0.07, p < .001, d = 0.30). Regarding age-related differences, we found small effect sizes that lower BRS was associated with increasing age, in that participants under 40 years old reported higher BRS scores than participants between 40 and 59 years old (M_Diff_ = .16, SE_M_ = 0.08, p = .044, d = 0.15) and older than 59 years (M_Diff_ = .27, SE_M_ = 0.09, p = .002, d = 0.24).

## Discussion

In this paper, we assessed the factor structure, reliability and validity of the German version of the BRS in a population-based sample of healthy participants and a representative sample from the German general population.

Our results provide evidence for a unidimensional structure of the six-item BRS once method effects due to the wording of the items were controlled for. This differs from previous research which either found a good fit obtained by a one-factor model [26,27] or a good fit obtained by two-factor model accounting for positively or negatively worded items [31]. However, based on our results, we do not recommend using positive and negative BRS sub scores and, thus, opted against this model since its multidimensionality can be explained by the artificial grouping of the positive and negative word of the items. Instead, we recommend using the unidimensional BRS score, although future research should find ways to reduce the method effects within the BRS.

With regard to reliability, our results (α = .85, ω = .85) are in line with results of the original validation study by Smith and colleagues (13) (α = .80 to .91), as well as the validation studies of the Spanish version (α = .83) (31), the Dutch version (α = .83), but lower than the α found in the validation of the Malaysian version (α = .93) [26] and higher than the α found for the Portuguese version (α = .76) [27] of the scale.

With regard to convergent validity, we also found negative correlations between the BRS score and symptoms of mental dysfunction (see Table 3). In addition, the correlation between the BRS score and social support found in our study was as reported by Smith and colleagues [13]. The same is true for the correlation between the BRS score and optimism. The findings of the present study could also confirm most of the correlations between the BRS and several coping subdomains. In contrast to Smith and colleagues [13], we did not find correlations between BRS scores and planning or behavioral disengagement. We did find negative correlations with venting and religion. Similar to previous studies [13,31], we also found a tendency for higher BRS scores for men. With regard to age, our findings partly contradict previous findings which report an increase of BRS scores with age [31,40]. A potential reason could be the age ranges of the study samples. Smith and colleagues [40] included two samples of undergraduate students (M = 20.56 and M = 21.09). In the sample of Rodríguez-Rey and colleagues [31], 87.20% were 50 years and younger. Our results may provide evidence that, when considering a larger age range, the ability to recover from stress decreases with increasing age. Compared to the study samples in the original paper, our participants were older than the two student samples (M = 20.04 and 19.80) and more within the range of the two clinical samples (M = 62.80 and 47.30). With regard to gender, there were slightly more men included in our samples compared to the healthy student samples in the original paper. The mean BRS score found in our study was well within the range reported by Smith and colleagues (13) (M = 3.53 to 3.98) for sample 1 (M = 3.58) and slightly lower for sample 2 (M = 3.37).

### Strengths and limitations

Advantages of our study are its population-based nature, the large sample sizes and the large age range that we were able to cover.

A potential limitation of our study may be the exclusion criteria applied in the GBS sample. The exclusion of subjects with known severe mental disorders, including subjects with substance dependence syndromes or users of prescribed psychoactive medications in the past six months may have caused a bias, since those subject groups are presumably less resilient. However, with regard to the BRS scores, the mean difference between the GBS samples (sample 1) and the representative sample (sample 2) is small (sample 1: M = 3.58, sample 2: M = 3.37; M_Diff sample 1/sample 2_ = .21). The results of the CFAs are similar for both samples. Therefore, we assume that the effect of the exclusion criteria in sample 1 should be minimal in our study.

Another potential limitation of our study compared to Smith and colleagues [13] is that we did not compare the BRS with other measures of resilience resources, such as the CD-RISC [12] or the Ego Resiliency Scale [18]. In addition, in both studies, the effect sizes found for coping subscales and social support are rather small. Although this indicates only a weak association with the BRS, the effects point in the same direction as found in the original paper.

## Conclusions and outlook

In contrast to other scales in the field of resilience research that mainly focus on potential resilience resources or resilience factors, the BRS measures the ability to bounce back or recover from stress itself [13]. As such, it looks more on the process of positive adaptation itself than on factors that may favor mental health despite adverse circumstances. The BRS can be applied in clinical practice with resilience interventions to examine the impact of an intervention on the ability to recover from stress. It could also be applied in research when studying resilience factors and underlying resilience mechanisms [2]. Due to its shortness, it can also be useful for epidemiological studies on mental health and psychological resilience in different populations or for studies which require brief and economic measures.

This study provides evidence for the reliability and validity of the German version of the BRS. In future studies, further confirmatory analyses should be conducted using the original scale published by Smith and colleagues [13], to assess whether the psychometric quality of the scale can be confirmed. Existing validation studies may benefit from reanalyzing their data to further test the factor structure. In addition, longitudinal research would allow assessing the potential of the BRS to predict dynamic changes in psychological resilience over time.

## Supporting information

S1 File German version of the Brief Resilience Scale. (PDF)
