# Supplementary material for: Population-based validation of a German version of the Brief Resilience Scale
Source: PLoS One. 2018 Feb 13;13(2):e0192761. doi: 10.1371/journal.pone.0192761 (PMC5811014; doi:10.1371/journal.pone.0192761)
Supplement: S1 File — (PDF) [file pone.0192761.s001.pdf]

S1 File. German version of the Brief Resilience Scale.

Geben Sie bitte an, wie sehr Sie den folgenden Aussagen jeweils zustimmen

	Stimme überhaupt nicht zu	Stimme eher nicht zu	Neutral	Stimme eher zu	Stimme vollkommen zu
1. Ich neige dazu, mich nach schwierigen Zeiten schnell zu erholen.	☐	☐	☐	☐	☐
2. Es fällt mir, schwer stressige Situationen durchzustehen.	☐	☐	☐	☐	☐
3. Ich brauche nicht viel Zeit, um mich von einem stressigen Ereignis zu erholen.	☐	☐	☐	☐	☐
4. Es fällt mir schwer zur Normalität zurückzukehren, wenn etwas Schlimmes passiert ist.	☐	☐	☐	☐	☐
5. Normalerweise überstehe ich schwierige Zeiten ohne größere Probleme.	☐	☐	☐	☐	☐
6. Ich brauche tendenziell lange, um über Rückschläge in meinem Leben hinwegzukommen.	☐	☐	☐	☐	☐
